# A Systematic Review of Social Media Use and Adolescent Identity Development

**DOI:** 10.1007/s40894-024-00251-1

**Published:** 2024-11-21

**Authors:** Hamide Avci, Laura Baams, Tina Kretschmer

**Affiliations:** Faculty of Behavioural and Social Sciences, Department of Pedagogy and Educational Sciences, University of Groningen, Grote Rozenstraat 38, Groningen, 9712 TJ The Netherlands

**Keywords:** Adolescents, Identity development, Self-concept, Identity distress, Social media

## Abstract

Social media have become a new context for adolescent identity development. However, it is challenging to build a thorough understanding of how social media and identity development are related because studies refer to different facets of social media engagement and use diverse concepts related to identity. This review synthesizes research on the relationships between quantity and quality of social media use and different dimensions of identity development, including identity exploration and commitment, self-concept clarity, and identity distress. The search conducted across four databases yielded 4,467 records, of which 32 studies were included in the analysis, comprising 19,658 adolescents with a mean age of 16.43 years (*SD* = 1.81) and an age range of eight to 26 years. Active participation in social media, rather than the amount of time spent on it, was associated with more identity exploration. Authenticity on social media, not idealized self-presentation, correlated with higher self-concept clarity. Additionally, adolescents who engaged in comparisons on social media demonstrated higher levels of identity exploration and identity distress. Overall, it seems to matter more for identity development what young people do on social media than how much time they spend on it.

## Introduction

Social media have become an integral part of adolescents’ daily lives who spend a significant amount of time online, often assuming dual roles as creators of self-generated content and consumers of content produced by others (Kolotouchkina et al., 2023). This engagement on social media platforms has attracted considerable research interest, including in what online activity means for identity development (e.g., Fullwood et al., 2016; Noon, 2020; Sebre & Miltuze, 2021). Unfortunately, a comprehensive understanding of existing findings on social media and its relationship to identity development is challenged by the lack of a unifying concept of social media engagement employed in research. Similarly, different identity concepts have been studied, which impedes straightforward conclusions about how engagement in social media and identity development are associated. Aiming to synthesize existing work, this study comprehensively reviews studies on the relationship between social media use and identity development by differentiating between quantity (e.g., time spent on social media) and quality (e.g., comparisons, self-presentation) of social media use, and by systematically exploring their associations with several important facets of identity development, namely commitment, exploration, self-concept clarity, and identity distress.

Adolescents form their identity through ongoing interactions between commitment and exploration. Commitments, which embody choices about one’s identity (Klimstra et al., 2010), are crucial not only for promoting identity certainty but also for fostering a coherent and stable self-concept. Self-concept clarity refers to a coherent and consistent self that is shaped by one’s committed values, beliefs, goals, and relationships and how these commitments integrate across different life domains (Schwartz et al., 2017). The alignment and stability provided by the integration of commitments are fundamental to promoting optimal adolescent development. For instance, commitments were linked to lower levels of anxiety and depression (Morsunbul et al., 2014), higher academic success (Pop et al., 2016), and self-concept clarity was associated with higher relationship satisfaction (Lewandowski Jr et al., 2010), positive self-esteem (Wu et al., 2010) and open communication with parents (Van Dijk et al., 2014). Moreover, if current commitments no longer adequately represent one’s identity, this can undermine self-concept clarity, leading to distress and potentially indicating the need for (further) identity exploration and revision (Schwartz et al., 2017).

Identity exploration involves a deep assessment of current commitments and questioning them to either revise or replace them with new ones. An analysis of current commitments is thought to be beneficial for maintaining one’s identity; whereas, reconsidering those commitments can potentially lead to identity uncertainty, which may have negative outcomes such as depressive and anxiety symptoms (Crocetti et al., 2008), lower academic achievement (Pop et al., 2016), and lower self-esteem (Crocetti et al., 2010). Furthermore, adolescents may experience identity distress, which can range from mild anxiety to severe symptoms in different life domains, such as long-term goals, friendships, and moral values, due to uncertainty about the self (Berman, 2020). Not unsurprisingly, individuals with identity distress reported higher levels of exploration (Berman et al., 2009) and lower levels of commitment (Sica et al., 2014). Identity distress has also been linked to internalizing and externalizing problems (Hernandez et al., 2006).

The process of identity development, which encompasses identity exploration and commitment and ideally results in a stable self-concept, with identity distress as the less favorable outcome, has been rooted in the relationships with family, peers, and school (Kroger, 2006), with research also largely focusing on these contexts. However, contemporary young people have expanded their social contexts through social media (Nesi et al., 2020). This expansion offers users various modes of interaction, either one-directional or multi-directional, where they can consume and create content, and receive feedback. Additionally, users can – to some extent –control and shape who has access to their content or profile. When studying effects of social media on adolescent development, researchers have primarily focused on measuring the amount of time young individuals spend on social media platforms. Time spent on social media has been linked to depression (Brunborg & Burdzovic Andreas, 2019), lower well-being (Huang, 2017), and a range of behavioral and emotional problems (Riehm et al., 2019). With respect to identity development, excessive time spent on social media was associated with low self-concept clarity (Sharif & Khanekharab, 2017) and disengagement from active identity exploration (Imperato et al., 2022). That said, findings are inconclusive and not all studies find associations between time spent on social media and identity exploration and commitment (e.g., Cyr et al., 2015; Fullwood et al., 2016). These mixed findings raise questions about the extent to which time spent on social media relates to facets of identity development and whether this conceptualization of social media engagement is sufficient.

In detail, engagement with social media comes in great diversity with feasibly differential associations with identity development but this is not captured when only time spent on social media is considered. This diversity includes various ways of self-presentation and seeking feedback on one’s presented persona, discussing concrete topics or sharing everyday issues, but also comparing abilities and opinions. Associations between forms – or quality – of engagement and facets of identity development have been studied and have shown, for instance, that individuals who compare their abilities on social media have lower self-concept clarity (Yang et al., 2018a) and higher identity distress (Yang et al., 2020) whereas editing profiles, sharing posts or blogs (Livingstone, 2008), and presenting oneself in different ways (Chittenden, 2010) or as someone else (Schmitt et al., 2008) are all indicative of identity exploration.

To date, no systematic review has addressed how different forms of social media engagement relate to adolescent identity development. Previous reviews suggested that social media enable identity exploration and various ways of self-presentation among adolescents (Shapiro & Margolin, 2014; Wängqvist & Frisén, 2016). However, these reviews addressed not only social media and personal identity development but also included other online contexts and group identities, which differs from the exclusive focus on social media and identity development in our current review. Finally, a recent systematic review examined the link between social media use and identity development (Senekal et al., 2023). Nonetheless, their conclusions might not fully capture this relationship, partly due to the broad approach in covering various concepts related to social media use (e.g., cyberbullying, online communication) and identity development (e.g., body image, self-esteem, self-concept clarity, and identity processing style).

## Current Study

Young people’s engagement on social media has quantitative and qualitative dimensions that are both associated with facets of identity development in different ways. However, this distinction has not been systematically applied in previous work. To overcome this limitation and to allow for a systematic understanding of how social media and identity development - both of crucial relevance for adolescent development - are related and what gaps in knowledge prevent comprehensive understanding, a synthesis of existing research is urgently needed. To this end, this systematic review of original qualitative and quantitative studies on adolescents’ social media engagement differentiates between quantity, that is time spent on special media and quality, that is, what young people do on social media, and identity development, including commitment and exploration, and its associated constructs self-concept and identity distress.

## Method

### Search Strategy

This review adhered to the updated reporting guidelines established by the Preferred Reporting Items for Systematic Review and Meta-Analysis (Page et al., 2021). The search was conducted in November 2022 on ERIC, MEDLINE, PsycINFO, and SocINDEX using the following terms: (identity) AND (“digital media” or internet or “social media” OR “social network” OR TikTok OR Snapchat OR Facebook OR YouTube OR weblog OR blog OR vlog OR Myspace OR Weibo OR Douyin OR QQ OR Kuashou OR Reddit OR Quora OR Instagram) AND (“young people” OR adolesc* OR teenagers OR “young adults” OR teen or youth OR student). Concrete platform names were selected based on their global popularity (Statista, 2022). Platforms that previously functioned as social media were also used as search terms to ensure that relevant studies published earlier were not overlooked. The search encompassed titles, abstracts, and keywords of articles. The search results were imported into Zotero to remove duplicate items, book chapters, dissertations, and conference studies. Subsequently, title and abstract screening were performed using Rayyan (Ouzzani et al., 2016).

### Eligibility Criteria

Studies were included if they fulfilled the following criteria: (1) pertained to adolescents with a mean age between nine and 19 years, or those with an age range from nine to 19; (2) assessed exploration and commitment, self-concept clarity, and identity distress as correlates of social media; (3) original research was qualitative or quantitative; (4) were published in a peer-reviewed journal; and (5) were in English.

Studies were excluded if they did not meet the inclusion criteria, including those in which: (1) participants were recruited based on a clinical diagnosis (e.g., depression, diabetes); (2) studies examined platforms initially developed purely as messenger services such as WhatsApp, Telegram, and WeChat; (3) studies assessed general internet use or utilization of any communication devices such as mobile phones, tablets, or computers; (4) studies examined online gambling, addiction, purchases, or learning; and (5) studies focused on social identities (e.g., sexual, religious, ethnic, gender, civic, national, or professional identities) instead of aspects of identity development, including commitment and exploration, self-concept clarity, and identity distress.

### Quality Assessment

The quality of eligible studies was assessed via assessment tools for qualitative and quantitative research. The National Institutes of Health Quality Assessment Tool for Observational Cohort and Cross-Sectional Studies was modified and used to evaluate the quality of quantitative studies. (NIH 2014). Specifically, our assessment included items related to study design, study population, sample size, independent and dependent variables, and follow-up rate. Other items were excluded because our systematic review did not include cohort studies. As a result, a quantitative study could receive a maximum score of six (best quality). The Critical Appraisal Skills Programme Qualitative Studies Checklist was used to assess the quality of qualitative studies (CASP 2018). Although the tool consists of ten items, two items concerning ethical issues and the value of the research were excluded, as these aspects were not considered when assessing the quality of the quantitative studies. Therefore, a qualitative study could receive a maximum score of eight (best quality).

## Results

### Data Extraction

The search generated *k* = 4,467 hits, all of which were loaded into Zotero. Duplicate items and non-peer-reviewed studies such as book chapters, dissertations, and conference studies were removed in Zotero, resulting in *k* = 2,685 for title and abstract screening. Following this, titles and abstracts were screened using Rayyan (Ouzzani et al., 2016), which resulted in *k* = 144 studies that remained for full-text screening.

**Fig. 1:**
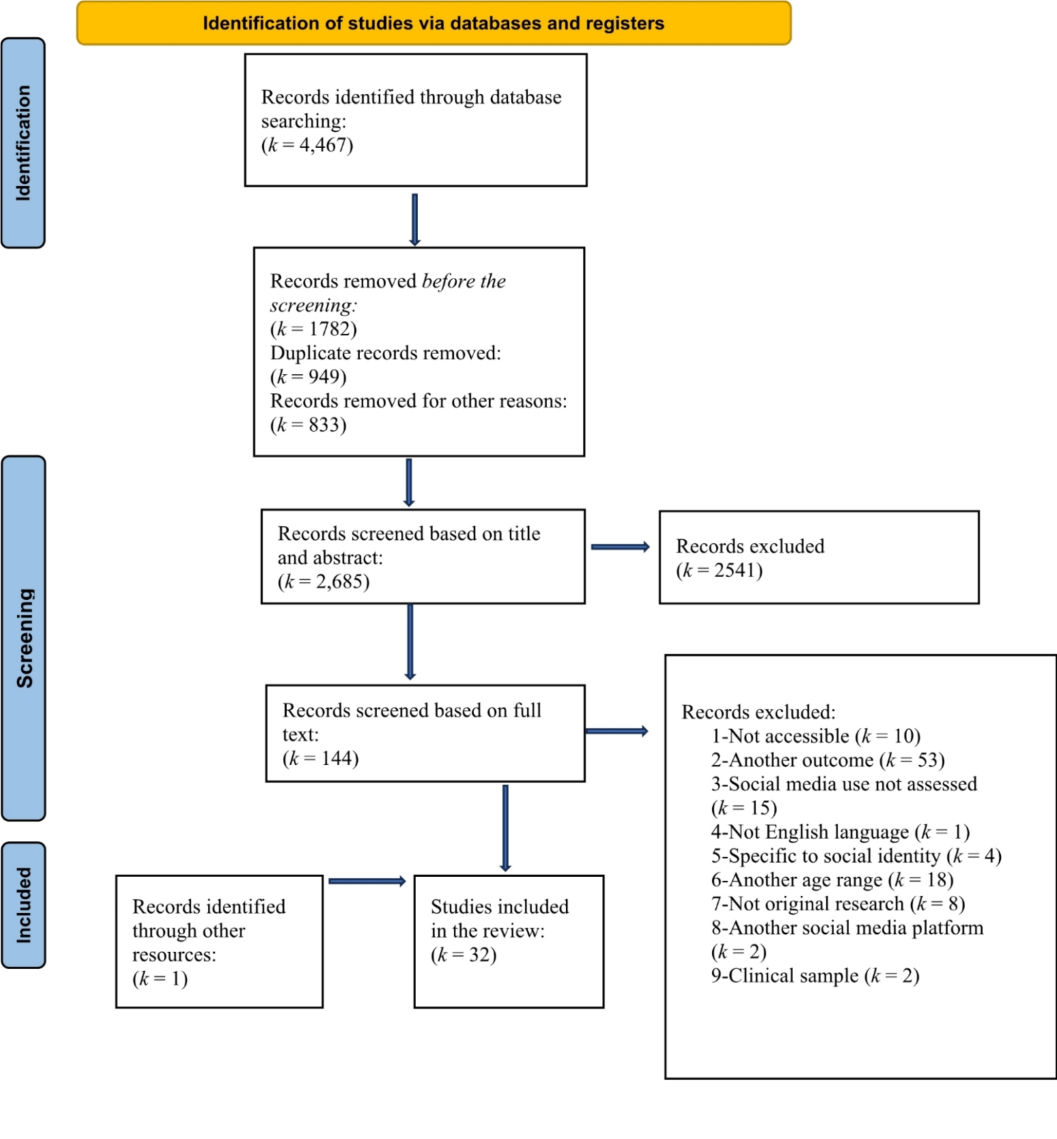
PRISMA flow diagram showing the process of study selection for inclusion in the systematic review

To ensure reliability, the second author screened 20% of randomly selected abstracts and titles, resulting in an 84% overlap with the first author’s selections. When authors disagreed, studies were discussed until consensus was reached. One study was identified through a reference cited in another study that was included for full-text screening. Ultimately, data were extracted from *k =* 32 out of *k =* 145 studies (see Fig. 1).

### Sample Characteristics

This review encompasses both qualitative *k* = 12 and quantitative *k* = 20 studies. The overall sample is *N* = 19,658 adolescents with a mean age of 16.43 years (*SD* = 1.81), ranging from eight to 26 years. The primary age range was set at nine to 19 years, but some studies included participants outside this range. However, the mean age in these studies was neither smaller than nine nor bigger than 19. The overall calculated mean age of 16.43 years (SD = 1.81) comes from studies that reported only the mean age of their participants.

The majority of studies were conducted in the US (*k* = 9), followed by the UK (*k* = 5), Singapore (*k* = 3), China (*k* = 2), Australia (*k* = 1), Malaysia (*k* = 1), Canada (*k* = 1), Ireland (*k* = 1), Malta (*k* = 1), Italy (*k* = 1), and Bermuda (*k* = 1). Four studies gathered data from multiple countries, and two studies did not report the country in which the studies were conducted (Guzzetti & Gamboa, 2005; Pelling & White, 2009).

### Summary of Included Studies

Details about sample sizes, measures of social media and identity development, design and analytic procedure of the studies are presented in the table (see Tables 1, 2, 3, 4, 5 and 6). The studies examined engagement on Facebook (*k* = 4), personal blogs/weblogs (*k* = 2), Instagram (*k* = 2), online journals (*k* = 1), Myspace (*k* = 1), and Ask.fm (*k* = 1). Thirteen studies did not name any social media platforms, eight included multiple platforms.

**Table 1:** Studies on identity commitment (k = 4)

Study	Country	Sample SiSSize	Sample Characteristics	Design	Method	Social Media Platforms	Social Media Measure	Identity Measure	Analysis Procedure and Findings
Cyr et al. (2015)	USA	*N* = 268	Mean age = 16.21 69% Female	CS	Pen and paper survey	Not specified	Technology Usage Scale (Cyr et al., 2015), developed for study purpose of study.	Ego Identity Process Questionnaire (Balistreri et al., 1995)	Correlation analysis revealed no significant association.
Imperato et al. (2022)	Italy	*N* = 354	Mean age = 16.18 *SD* = 1.58 80% Female	CS	Pen and paper survey	Not specified	Internet Addiction (Ferraro et al., 2007), adapted for purpose of study, and adolescents’ self-reports on time spent on social media.	Utrecht Management of Identity Commitments Scale (Crocetti et al., 2010)	Correlation analysis revealed that both of the two tested associations were significantly negative.
Noon (2020)	UK	*N* = 177	Mean age = 15.45 *SD* = 1.67 55% Female	CS	Pen and paper survey	Instagram	Social Media Social Comparison Scale (Yang et al., 2018a)	Utrecht Management of Identity Commitments Scale (Crocetti et al., 2010)	Multivariate multiple regression revealed one of the two tested associations was significantly positive.
Noon et al. (2021)	Romania and Serbia	*N* = 1085	Mean age = 18.87 *SD* = 2.57 78% Female	CS	Not reported	Instagram	Social Media Social Comparison Scale (Yang et al., 2018a)	Utrecht Management of Identity Commitments Scale (Crocetti et al., 2010)	Hierarchical regression analysis revealed that one of the two tested associations was significantly positive.

Abbreviations: CS: cross-sectional

**Table 2:** Quantitative studies on exploration (k = 6)

Study	Country	Sample Size Sie	Sample Characteristics	Design	Method	Social Media Platforms	Social Media Measure	Identity Measure	Analysis Procedure and Findings
Cyr et al. (2015)	USA	*N* = 268	Mean age = 16.21 69% Female	CS	Pen and paper survey	Not specified	Technology Usage Scale (Cyr et al., 2015), developed for study.	Ego Identity Process Questionnaire (Balistreri et al., 1995)	Correlation analyses revealed no significant associations.
Imperato et al. (2022)	Italy	*N* = 354	Mean age = 16.18 *SD* = 1.58 80% Female	CS	Pen and paper survey	Not specified	Internet Addiction Test (Ferraro et al., 2007), adapted for purpose of study, and adolescents’ self-reports on time spent on social media.	Utrecht Management of Identity Commitments Scale (Crocetti et al., 2010)	Correlation analyses revealed that one of the four tested associations was significantly negative, while the other was significantly positive.
Manzi et al. (2018)	Italy and Chile	*N* = 712	Mean age = 16.40 *SD* = 2.03 52% Female	CS	Pen and paper survey	Facebook	Facebook use was measured by two items.	Identity exploration was measured by two items.	Correlation analysis indicated that six of the eight tested associations were significantly positive.
Noon (2020)	UK	*N* = 177	Mean age = 15.45 *SD* = 1.67 55% Female	CS	Pen and paper survey	Instagram	Social Media Social Comparison Scale (Yang et al., 2018a)	Utrecht Management of Identity Commitments Scale (Crocetti et al., 2010)	Multivariate multiple regression revealed that three of the four tested associations were significantly positive.
Noon et al. (2021)	Romania and Serbia	*N* = 1085	Mean age = 18.87 *SD* = 2.57 78% Female	CS	Not reported	Instagram	Social Media Social Comparison Scale (Yang et al., 2018a)	Utrecht Management Of Identity Commitments Scale (Crocetti et al., 2010)	Hierarchical regression analysis revealed that three of the four tested associations were significantly positive.
Schmitt et al. (2008)	USA	*N* = 500	Aged 8–17	CS	Phone interview	Personal homepages and blogs	A survey questionery on technology use.	A survey questionery about self presentation and online interaction.	ANOVA revealed that young people who created homepages were more likely to pretend to be someone other than they were, compared to those who did not create homepages.

Abbreviations: CS: cross-sectional

**Table 3:** Qualitative studies on identity exploration (k = 6)

Study	Country	Sample Size	Sample Characteristics	Method	Social Media Platforms	Analysis Procedure and Findings
Chittenden (2010)	USA, Canada, New Zealand	*N* = 10	Aged 14–18 100% Female	Open- ended questions asked via email,	Fashion blogs	Qualitative data analysis revealed that fashion blogs allow adolescents to present themselves by playing different looks and styles as part of their identity exploration.
Farrugia et al. (2019)	Malta	*N* = 22	Aged 11–16 45.45% Female	Focus group discussion and interviews	Ask.fm	Thematic analysis revealed that adolescents engage in self-presentation on Ask.fm by answering questions and reflecting on feedback, which helps them explore their identities.
Greenhow and Robelia (2009)	USA	*N* = 11	Aged 17–19 72.73% Female	Semi-structured interview, Think aloud, Content analysis,	MySpace	The thematic analysis revealed that MySpace was a platform for young people to try out different roles and play with their profile looks as part of their identity exploration.
Kennedy and Lynch (2016)	Ireland	*N* = 16	Aged 9–16 69% Female	Focus group interview	Not specified	Thematic analyses revealed that adolescents presented themselves by creating fake ages or avatars and presenting their edited versions to explore their identities.
Lichy et al. (2022)	UK	*N* = 16	Aged 8–12 50% Male	Interview	Not specified	The data were analysed using axial coding. Social media platforms provide an environment for presenting oneself as someone else, facilitating identity exploration.
Livingstone (2008)	UK	*N* = 16	Aged 13–16 50% Female	Open ended individual interviews	MySpace, Facebook, Bebo, Piczo	Thematic analyses revealed that social media helps individuals explore their identity by providing flexibility to revise and edit their profiles.
Mazur and Kozarian (2010)	USA	Not applicable	124 blogs were examined, each of which was owned by individuals aged between 15 and 19	Content analyses	LiveJournal, DeadJournal, Xanga, Open Diary, Myspace, Friendster	Data were analyzed by thematic analysis. Blog entries reflected confusion of the authors about themselves, which was 7% in all entiries. Blogs were a place where adolescents explored who they were and who they wanted to become.

**Table 4:** Quantitative studies on self-concept clarity (k = 12)

Study		Country	Sample SiSSize	Sample Characteristics	Design	Method	Social Media Platforms	Social Media Measure	Identity Measure	Analysis Procedure and Findings
Davis (2013)	Bermuda		*N* = 2079	Mean age = 15.4 57% Female	CS	Pen and paper, digital survey	Facebook Youtube	Digital media use scales (e.g., Courtois et al., 2009; Leung, 2007), adapted for purpose of study.	Self-Concept Clarity Scale (Campbell et al., 1996)	Structural equation model indicated that one tested association was significantly negative.
Fullwood et al. (2016)	UK		*N* = 148	Mean age = 15.50 *SD* = 1.87 61% Femaale	CS	Pen and paper survey	Facebook	Facebook Intensity Scale (Ellison et al., 2007) Online Self Scale (Fullwood et al., 2016) developed for purpose of study.	Self-Concept Clarity Scale (Campbell et al., 1996)	Correlation analysis of five tested associations indicated no association for two, two were significantly negative, and one was significantly positive.
Ho et al. (2017)	Singapore		*N* = 4920	Mean age = 14.41 *SD* = 2.72 57% Female	CS	Pen and paper survey	Not specified	Excessive Social Media Use Scale (Caplan, 2010), adapted for purpose of study.	Self Identity Scale (Callero, 1985), adapted for purpose of study.	Hierarchical regression analysis revealed one tested association was significantly positive.
Lee et al. (2017)	Singapore		*N* = 4920	Mean age = 14.73 51% Female	CS	Pen and paper survey	Not specified	SNS Habit Strength Scale (Larose et al., 2003), adapted for purpose of study.	Self Identity Scale (Callero, 1985), adapted for purpose of study.	Covariance structure model revealed that one tested association was significantly positive.
Pelling & White (2009)	Not reported		*N* = 233	Mean age = 19.22 *SD* = 2.03 64% Female	CS	Both online and pen and pencil survey	Not specified	Self-report of participants on how often they visit social networking sites.	Self Identity Scale (Terry et al., 1999), adapted for purpose of study.	Hierarchical regression analysis indicated that one tested association was significantly positive.
Sharif & Khanekharab (2017)	Malaysia		*N* = 501	Mean age = 19.68 *SD* = 1.65 56% Female	CS	Online survey	Not specified	Excessive SNS Usage Scale (Mueller et al., 2011), adapted for purpose of study.	Self-Concept Clarity Scale (Campbell et al., 1996)	The structural equation model revealed that one tested association was significantly positive.
Yang and Brown (2016)	USA		*N* = 218	Mean age = 18.07 *SD* = 0.33 64% Female	L	Online survey	Facebook	Audience Supportive Feedback Scale (Yang & Brown, 2016) developed for purpose of study.	Self-Concept Clarity Scale (Campbell et al., 1996)	Path analysis revealed no significant associations.
Yang et al. (2017)	USA		*N* = 219	Mean Age = 18.29 *SD* = 0.75 74% Female	CS	Online survey	Instagram, Twitter, Snapchat, Facebook, Other	Facebook self-presentation scale (Yang & Brown, 2016), adapted for purpose of study.	Erikson Psychosocial Stage Inventory (Rosenthal et al., 1981)	Correlation analysis of the four tested associations indicated no association for one, one was significantly negative and two were significantly positive.
Yang et al. (2018a)	USA		*N* = 219	Mean age = 18.29 *SD* = 0.75 74% Female	L	Online survey	Instagram, Twitter, Snapchat, Facebook, Other	Social Media Social Comparison Scale (Yang et al., 2018a), adapted for purpose of study.	Erikson Psychosocial Stage Inventory (Rosenthal et al.1981)	Correlation analysis indicated five of eight tested associations were significantly negative.
Yu and Shek (2021)	China		*N* = 1896	Mage = 13.19 52% Male	CS	Pen and paper survey	Not specified	Bergen Social Media Addiction Scale (e.g., Andreassen et al., 2017; Lin et al., 2017), adapted for purpose of study.	Chinese Positive Youth Development Scale (Shek et al., 2007)	Correlation analysis indicated that one tested association was significantly negative.
Wang et al. (2021)	China		*N* = 473	Mean age = 14.63 *SD* = 1.57 61% Male	CS	Pen and paper survey	Not specified	Adolescents’ Mobile Social Media Usage Behaviour Scale (Wang & Lei 2015), adapted for purpose of study.	Self-Identity Scale (Tan et al., 1977)	Correlation analysis indicated one tested association was significantly positive.
Zheng et al. (2011)	Singapore		*N* = 101	Mean age = 14.77 *SD* = 1.47 73% Female	CS	Online survey	MysSpace Facebook Other	Demographic Information Questionnaire	Adolescent Online Social Communication Questionnaire	Hierarchical regression analysis revealed no association.

Abbreviations: CS: cross-sectional; L: longitudinal

**Table 5:** Qualitative studies on self-concept clarity (k = 6)

Study	Country	Sample Size	Sample Characteristics	Method	Social Media Platforms	Analysis Procedure and Findings
Bell (2019)	UK	*N* = 35	Aged 13–17 60% Female	Semi-structured interview and focus group discussion	Not specified	Data were analyzed by thematic analysis. The amount of likes that adolescents received on their social media content as a way of self-presentation reinforced their self-concept.
Guzzetti & Gamboa, 2005	Not reported	*N* = 2	Age not reported/ adolescents 100% Female	Semi-structured interview, observations, and open-ended response questionnaire	Live Journal	Data were analyzed using constant comparison. Online journaling, with features like immediate feedback and shared reflections, can reciprocally shape and reinforce one’s self-concept.
Pangrazio (2019)	Australia	*N* = 13	Aged 15–19	Interviews, online questionnaire, online observations, and group discussions on workshops	Facebook	Data were analyzed through thematic analysis. Facebook is a place where adolescents present themselves; however, when they present their edited versions, it creates confusion about who they are and who they present.
Richardson (2016)	Canada	*N* = 150	Not reported/ adolescents	Online Survey Focus Group Study, Interview	Not specified	Data were analysed by thematic analysis. Presenting their true self on social media reinforced adolescents’ self-concepts, while false presentation created confusion.
Schmitt et al. (2008)	USA	Not applicable	48 blogs were examined, each of which was owned by individuals aged between 8 and 17	Qualitative content analyses	Personal homepages and Blogs	Data were analysed by thematic analysis. The home page is a space where adolescents present and express themselves, which facilitates their self-concept.
Subrahmanyam et al. (2009)	US, Canada, Australia, UK, Singapore, Brunei, Indonesia, Malaysia, New Zealand,	Not applicable	195 blogs were examined, each of which was owned by individuals aged between 14 and 18	Qualitative content analyses	Xanga, LiveJournal, Blog-City, Blog Drive, Journal Space, Blogsearchengine.com, Blurty, DeadJournal, Open Diary	Data were analysed by thematic analysis. Only 10% of the entries addressed teenagers’ self-concept. Blogs can help reinforce an individual’s self-concept by allowing them to present themselves in the blogosphere and receive feedback.

**Table 6:** Studies on identity distress (k = 3)

Study	Country	Sample Size Siz	Sample Characteristics	Design	Method	Social Media Platforms	Social Media Measure	Identity Measure	Analysis Procedure and Findings
Cyr et al. (2015)	USA	*N* = 268	Mean age = 16.21 69% Female	CS	Pen and paper survey	Social networking sites	Technology Usage Scale (Cyr et al., 2015b), developed for study purpose of study.	Identity Distress Survey (Berman et al., 2004)	Correlation analysis indicated one tested association was significantly negative.
Yang et al. (2018b)	USA	*N* = 219	Mean age = 18.29 *SD* = 0.75 74% Female	L	Online survey	Instagram Twitter Snapchat Facebook Other	Social Media Social Comparison Scale (Yang et al., 2018a)	Identity Distress Survey (Berman et al., 2004)	Correlation analysis indicated two of the four tested associations were significantly positive.
Yang et al. (2020)	USA	*N* = 219	Mean Age = 18.29 *SD* = 0.75 74% Female	L	Not reported	Not specified	Social Media Social Comparison Scale (Yang et al., 2018a)	Identity Distress Survey (Berman et al., 2004)	Correlation analysis revealed one of the three tested associations was significantly positive.

Abbreviations: CS: cross-sectional; L: longitudinal

Studies examined the link between social media engagement quantity (i.e., how much time someone had spent on social media), commitment (*k* = 2), exploration (*k* = 2), self-concept clarity (*k* = 6), and identity distress (*k* = 2). Associations between social media engagement quality (i.e., what adolescents do on social media), and aspects of identity development were also assessed, concerning commitment (*k* = 2), exploration (*k* = 10), self-concept clarity (*k* = 13), and identity distress (*k* = 1).

Studies employed various instruments, including newly developed or modified scales, to measure time spent on social media. Further, adolescents were asked to self-report the amount of time they spent on social media platforms. To assess social comparison on social media as a form of engagement, all studies employed the Social Media Social Comparison Scale (Yang et al., 2018a) (*k* = 5). Self-presentation strategies and receiving feedback on social media were measured using various methods in quantitative studies and through interviews, content analysis, and focus group discussions.

Regarding outcomes, commitment and exploration were measured by the Ego Identity Process Questionnaire in one study (Balistreri et al., 1995), but the most frequently used scale wa*s* the Utrecht Management of Identity Commitments Scale (Crocetti et al., 2010) (*k* = 3). Self-concept clarity was assessed by various instruments, with the most used being the Self-Concept Clarity Scale (Campbell et al., 1996) (*k* = 4). All studies that measured identity distress used the Identity Distress Survey (Berman et al., 2004) (*k* = 3).

### Quality Assessment

Regarding the quality assessment of quantitative studies, none received a score on the item concerning the follow-up rate as the majority of the studies were cross-sectional, and the longitudinal studies had a loss to follow-up rate higher than 20%; thus, none achieved the maximum score of six. Only one study justified its sample size (Davis, 2013); hence, the remaining studies did not receive points on this item. All studies provided their sample information and formulated a research question. Three studies defined identity development as a part of being a social media user (Ho et al., 2017; Lee et al., 2017; Pelling & White, 2009), which might bias the findings by over-attributing identity development to social media use. Additionally, while one study did not clearly define identity development (Schmitt et al., 2008), two studies did not explicitly conceptualize social media use (Pelling & White, 2009; Schmitt et al., 2008). Despite the limitations of included studies, the overall quality was considered satisfactory (see Table 7).

**Table 7:** Quality assessment of included quantitative studies using the national institutes of health quality assessment tool for observational cohort and cross-sectional studies

Study	Was the objective clearly or research question clearly stated?	Was the study population clearly specified and defined?	Was sample size justification, power description, or variance and effect estimate provided?	Were the social media variables clearly defined, valid, reliable, and implemented consistently across all study participants?	Were identity development variables clearly defined, valid, reliable, and implemented consistently across all study participants?	Was loss to follow-up after baseline 20% or less?	Quality rating
(Cyr et al., 2015)	1	1	0	1	1	0	4
(Davis, 2013)	1	1	1	1	1	0	5
(Fullwood et al., 2016)	1	1	0	1	1	0	4
(Ho et al., 2017)	1	1	0	1	0	0	3
(Imperato et al., 2022)	1	1	0	1	1	0	4
(Lee et al., 2017)	1	1	0	1	0	0	3
(Manzi et al., 2018)	1	1	0	1	1	0	4
(Noon, 2020)	1	1	0	1	1	0	4
(Noon et al., 2021)	1	1	0	1	1	0	4
(Pelling & White, 2009)	1	1	0	0	0	0	2
(Schmitt et al., 2008)	1	1	0	0	0	0	2
(Sharif & Khanekharab, 2017)	1	1	0	1	1	0	4
(Wang et al., 2021)	1	1	0	1	1	0	4
(Yang & Brown, 2016)	1	1	0	1	1	0	4
(Yang et al., 2017)	1	1	0	1	1	0	4
(Yang et al., 2018a)	1	1	0	1	1	0	4
(Yang et al., 2018b)	1	1	0	1	1	0	4
(Yang et al., 2020)	1	1	0	1	1	0	4
(Yu & Shek, 2021)	1	1	0	1	1	0	4
(Zheng et al., 2011)	1	1	0	1	1	0	4

None of the qualitative studies received a score of eight, for different reasons, such as lack of rigorous data analysis, reporting of appropriateness of research design, and the relationship between researcher and participants. All studies explained the appropriateness of their qualitative research methodology and recruitment strategies, whereas one study failed to detail its recruitment approach (Richardson, 2016). Two studies did not clearly articulate their research questions (Lichy et al., 2022; Richardson, 2016). The majority of the studies did not address the relationship between researchers and participants, except for two (Chittenden, 2010; Guzzetti & Gamboa, 2005). While the majority provided depth description of data analysis process, three did not (Chittenden, 2010; Lichy et al., 2022; Livingstone, 2008). Only six studies explicitly stated their results. Notwithstanding the limitations of the studies, the overall quality was deemed satisfactory (see Table 8).

**Table 8:** Quality assessment of included quantitative studies using the critical appraisal skills programme qualitative studies checklist

Study	Was there a clear statement of the aims of the research?	Is a qualitative methodology appropriate?	Was the research design appropriate to address the aim of the research?	Was the recruitment strategy appropriate to aims of the research?	Was the data collected in a way that addressed the research issue?	Has the relationship between researcher and participants been adequately considered?	Was the data analysis sufficiently rigorous?	Is there a clear statement of findings?	Quality Rating
(Bell, 2019)	1	1	1	1	1	0	1	0	6
(Chittenden, 2010)	1	1	0	1	1	1	0	0	5
(Farrugia et al., 2019)	1	1	0	1	1	0	1	1	6
(Greenhow & Robelia, 2009)	1	1	1	1	1	0	1	1	7
(Guzzetti & Gamboa, 2005)	1	1	1	1	1	1	1	0	7
(Kennedy & Lynch, 2016)	1	1	0	1	1	0	1	1	6
(Lichy et al., 2022)	0	1	0	1	1	0	0	0	3
(Livingstone, 2008)	1	1	0	1	1	0	0	1	5
(Mazur & Kozarian, 2010)	1	1	1	1	1	0	1	1	7
(Pangrazio, 2019)	1	1	1	1	1	0	1	1	7
(Richardson, 2016)	0	1	0	0	1	0	1	0	3
(Schmitt et al., 2008)	1	1	1	1	1	0	1	0	6
(Subrahmanyam et al., 2009)	1	1	1	1	1	0	1	0	6

### Social Media Use and Commitment

Two studies examined the association between time spent on social media and identity commitment, with mixed findings. One study revealed no significant association between time spent on social media and commitment (Cyr et al., 2015), while another study indicated that adolescents who spent excessive or normal amount of time on social media tended to quit their commitments (Imperato et al., 2022).

Two studies focused on comparisons on social media and identity commitments. While one study reported that adolescents who compared their abilities were more certain about themselves (Noon, 2020), another study indicated that those who compared opinions were more confident about who they were (Noon et al., 2021). The age differences between these two studies could explain this variance; the first study involved younger adolescents, while the second included older adolescents. This suggests that while younger adolescents may present themselves as capable, older adolescents may present themselves as knowledgeable. Nevertheless, a general conclusion on the link between the comparison of abilities and opinions on social media and identity commitment cannot be drawn due to the cross-sectional nature of the research and the limited number of studies.

### Social Media Use and Exploration

Two studies investigated the link between time spent on social media and identity exploration, yielding mixed outcomes that precluded a conclusive finding. While one study found no association between time spent on social media and exploration (Cyr et al., 2015), another study reported that individuals who spent excessive amounts of time on social media were more likely to have identity uncertainty (Imperato et al., 2022).

Six studies examined the relationship between different ways of self-presentation and identity exploration. Studies suggested that adolescents presenting themselves by playing with different looks (Chittenden, 2010), testing different roles (Greenhow & Robelia, 2009) and answering questions about who they were (Farrugia et al., 2019), and pretending to be someone else (Kennedy & Lynch, 2016; Lichy et al., 2022; Schmitt et al., 2008) tended to explore their identities. Four studies suggested that adolescents who edited their profiles, shared posts or content, and created blog entries were more likely to engage in identity exploration (Greenhow & Robelia, 2009; Livingstone, 2008; Manzi et al., 2018; Mazur & Kozarian, 2010). In summary, social media use seems to be important for identity exploration among those who actively participate.

Two studies examined the relationship between the comparison of abilities and opinions and exploration. One study showed that adolescents who compared their abilities were more likely to analyze and reconsider those abilities (Noon, 2020), while another study found that those who compared their opinions analyzed and questioned those opinions (Noon et al., 2021). In summary, ability and opinion comparison may be associated with identity confusion, which is also beneficial for engaging in further exploration and making new commitments. Unfortunately, due to the cross-sectional nature of the research and the small number of studies, it is difficult to reach a broad conclusion on this relationship.

### Social Media Use and Self-Concept Clarity

Six studies addressed time spent on social media and self-concept clarity, with mixed results. Two studies found no significant correlation between time spent on social media and self-concept clarity (Fullwood et al., 2016; Zheng et al., 2011). In contrast, two studies indicated that adolescents who used social media excessively had an unclear self-concept (Sharif & Khanekharab, 2017; Yu & Shek, 2021). Two studies found that those who spent excessive time on social media had a clear self-concept (Ho et al., 2017; Pelling & White, 2009). Given the mixed results, it is difficult to draw definitive conclusions about the relationship between time spent on social media and self-concept clarity.

A short-term longitudinal study examined the correlation between comparison on social media and self-concept clarity, showing that individuals who compared their abilities tended to feel unclear about themselves, as evidenced by both longitudinal and cross-sectional associations. There was a negative relationship between opinion comparison and self-concept clarity when measures were taken approximately five months after the initial assessment (Yang et al., 2018a). This suggests that both forms of comparison are related to unclear self-concepts, with opinion comparisons possibly occurring after ability comparisons. It is difficult to make a general statement about this relationship as the number of studies is limited.

Four studies showed that adolescents who presented their true selves in a way that matched their in-person version had a clear self-concept (Fullwood et al., 2016; Richardson, 2016; Schmitt et al., 2008; Yang et al., 2017). In contrast, those who presented an idealized self that was inconsistent with their offline persona were less clear about how they felt about themselves (Davis, 2013; Fullwood et al., 2016; Pangrazio, 2019; Richardson, 2016; Yang et al., 2017). The evidence indicates that when individuals present themselves in a manner that aligns with their authentic selves, it fosters self-concept clarity.

Two studies showed that adolescents who regularly used social media, shared content, and engaged in recreational behaviors were more likely to be sure who they were (Lee et al., 2017; Wang et al., 2021). Four studies focused on the relationship between getting feedback on social media and self-concept clarity, yielding mixed results. Three qualitative studies found that those who got feedback from their audience reinforced their self-concept clarity (Bell, 2019; Guzzetti & Gamboa, 2005; Subrahmanyam et al., 2009). Nevertheless, one short-term longitudinal study did not reveal a significant correlation between getting feedback on social media and self-concept clarity ( Yang & Brown, 2016).

### Social Media Use and Identity Distress

Three studies examined the link between social media use and identity distress. One study found that people who spent time on social media had identity-related distress (Cyr et al., 2015), whereas another study did not reveal any significant relationship, either cross-sectional or longitudinal, between excessive use of social media and identity distress (Yang et al., 2020). Only one short-term longitudinal study reported a cross-sectional association; people who compared their abilities were likely to experience identity distress (Yang et al., 2018b). Consequently, establishing a definitive relationship between time spent on social media and identity distress is difficult due to mixed results. Nevertheless, ability comparison may be a potential correlate of identity distress.

## Discussion

Social media have become an indispensable part of adolescents’ lives, and the relationship between social media engagement and adolescent development, including identity development, has been the focus of many studies. However, a comprehensive understanding of the interplay between social media and identity development is challenging due to heterogeneity in conceptualizing and assessing social media use and the different facets of identity development. Therefore, the empirical literature was reviewed to identify whether systematic associations between aspects of social media engagement and identity development can be derived, with a specific focus on associations between the quantity and quality of social media engagement and components of identity development, including commitment and exploration, self-concept clarity, and identity distress.

Overall, our synthesis of the research suggests that it is *not* the amount of time spent on social media that is critical, but rather the activities undertaken during this time that seem to play a role for aspects of identity development. Our findings are in line with findings that appearance comparison on social media was associated with body dissatisfaction (Fioravanti et al., 2022), and particular types of engagement, such as comparisons, active or passive use, were associated with symptoms of depression, anxiety, and psychological distress (Keles et al., 2020), where actual activities were more salient than the amount of time spent engaging in social media. For example, an adolescent interested in science might reinforce their identity by watching videos about chemistry on platforms such as YouTube. Such passive engagement in consuming content is simultaneously an active process of identity exploration and reinforcement for the individual, or vice versa. Simply quantifying screen time or time spent on social media does not adequately capture the nuances of social media use. By understanding what users are doing on these platforms, deeper insights into how social media use relates to facets of identity development are possible.

Our findings also suggest that self-presentation strategies are common among adolescents on social media. These strategies include playing with appearance to present an ideal self, impersonating others, and creating fictitious profiles, all of which can be part of one’s identity exploration (Chittenden, 2010; Greenhow & Robelia, 2009; Kennedy & Lynch, 2016; Schmitt et al., 2008) That said, taking up different identities in social media contexts might also hinder achieving self-concept clarity. That is, adolescents who present a fictitious version of themselves that differs from their authentic self tend to have lower self-concept clarity (Davis, 2013; Fullwood et al., 2016; Pangrazio, 2019; Richardson, 2016; Yang et al., 2017). In contrast, adolescents who present their true selves on social media tend to have a clear self-concept (Fullwood et al., 2016; Schmitt et al., 2008; Richardson, 2016; Yang et al., 2017), suggesting stronger commitments. As conclusions about a direction of effects cannot be drawn, it might also be the case that those with clearer self-concept are more confident to be who they actually are on social media. To fully understand adolescents’ self-presentation strategies on social media and their relation to identity development, it would be informative to examine adolescents’ motives for social media use because the reasons why one engages in fictitious self-presentation might vary. Adolescents may create alternate profiles in which they pretend to be older to overcome age restrictions, to bully without being recognized in the real world, or simply for amusement. In other words, presenting oneself in a manipulated way on social media may go beyond mere identity exploration, while presenting one’s true self is an indication of strong commitments.

Research on the relationship between comparing abilities and opinions on social media and aspects of identity development is limited, and our review includes only a few studies, suggesting some possible age effects. Younger adolescents who compared their abilities tended to re-evaluate their identity commitments in order to make new commitments or strengthen existing ones (Noon, 2020). Older adolescents experienced this when they compared their opinions (Noon et al., 2021). In other words, younger adolescents were more likely to present themselves by their abilities, while older adolescents tended to define themselves through their opinions on social media. However, firm temporal conclusions cannot be drawn due to the cross-sectional nature of these studies, which prevents observing changes over time.

A few limitations were encountered while conducting this review. Considering the field of identity development, most of the research has focused on a single aspect of identity development in the context of social media. For example, while many of the studies included in this review focus on exploration (*k* = 12), only few studies focus on commitment (*k* = 4). Given this narrow focus of current research, it remains unclear whether exploration ultimately results in commitment and what role social media plays in that regard. Future research should examine identity development as a whole process in the context of social media. Furthermore, the terms identity, *self*,* self-identity*,* and self-concept* have been used interchangeably, leading to conceptual ambiguity. It is important to distinguish that self or self-concept encompasses different identities in different domains and should not be considered synonymous with identity in future research.

In the context of social media research, the amount of time individuals spend on these platforms has been categorized using terms such as “excessive,” “problematic,” or “compulsive.” However, it is important to recognize that a higher quantity of time spent, based on individuals’ self-reports, on social media does not inherently indicate problematic use (O’Day & Heimberg, 2021). Similarly, the frequency of checking social media accounts does not necessarily equate to compulsive behavior. Determining what constitutes “excessive” use of social media might vary according to individual circumstances. It is recommended that future research should shift its focus from quantifying time spent on social media to examining the specific activities that adolescents are engaging in on these platforms. Understanding the nature and context of these activities will provide a more nuanced and comprehensive perspective on what is usual, excessive, or problematic use of social media.

Current research has largely focused on mainstream social media platforms that were popular in the late 2000s, throughout the 2010s and in early 2020s such as Facebook and Instagram. However, there is a significant shift towards newer platforms among young people, and the popularity and use of each platform change more rapidly than in the past. These newer platforms offer different opportunities for engagement. For example, young people on TikTok participate in video challenges, a mode of interaction that is not common on older platforms (Ahlse et al., 2020). YouTube offers extensive educational content (Orús et al., 2016), which can reinforce young people’s identities within educational domains. BeReal encourages users to share real-time, unedited photos to promote authenticity (Siepen, 2023), while Snapchat is known for its variety of filters that allow users to present an idealized self (Cruz, 2019). Although these platforms share some characteristics, each offers unique opportunities for engagement. As a result, it is important to update social media research frequently to track trends among adolescents and the new forms of interactions on the platforms and understand the impact of those on adolescent identity development.

In addition to the limitations of the field, this review has its own constraints. First, the studies included in this review are predominantly cross-sectional, which prevents tracking developmental processes over time and establishing a causal relationship between social media use and identity development. Furthermore, the scope of this review is limited by the specific search terms used, the databases selected, and the time frame for article retrieval, which may have excluded relevant studies. Only studies published in English were included, excluding potentially important articles in other languages as well as unpublished dissertations and book chapters. Finally, the quality assessment criteria were adapted for this review, so it is not possible to directly compare the quality scores with those of other studies.

## Conclusion

This systematic review aimed to enrich the field of identity development research by synthesizing existing evidence on the role of social media use in adolescent identity development. Taken together, it is apparent that it is the activities adolescents engage in on social media that are associated with identity development, not the amount of time spent on social media. Further, authentic self-presentation is more common among adolescents with greater self-concept clarity, while fictitious self-presentation is related to identity exploration. Unfortunately, given the cross-sectional nature of most studies, it remains unclear whether social media use affects identity development in adolescence, whether the adolescent identity development process influences social media use, or whether these associations are perhaps bidirectional. Consequently, there is a need for well-designed longitudinal studies to capture the temporal direction of this relationship. Lastly, measuring only the time spent on social media limits the understanding of the relationship between social media engagement and identity development. Future research should focus on adolescents’ activities, interactions, and the content they engage with on social media to provide a more complete picture of this relationship.

## Declarations

### Declaration of the Use of Generative AI and AI-Assisted Technologies

During the preparation of this work, HA used Chat GPT 4.0 to assist with editing grammar and correcting spelling mistakes. After using this tool, LB and TK reviewed and edited the content as needed, and the authors take full responsibility for the content of the publication.
